# Ventilation challenge in rigid bronchoscopy: Laser tube as an alternative management in patients with lung cancer and central airway obstruction

**DOI:** 10.1111/1759-7714.14671

**Published:** 2022-11-23

**Authors:** Gaetana Messina, Mary Bove, Giovanni Natale, Vincenzo Di Filippo, Giorgia Opromolla, Eva Massimilla, Mauro Forte, Anna Rainone, Giuseppe Vicario, Beatrice Leonardi, Alfonso Fiorelli, Giovanni Vicidomini, Mario Santini, Mario Pirozzi, Marianna Caterino, Carminia Maria Della Corte, Fortunato Ciardiello, Morena Fasano

**Affiliations:** ^1^ Thoracic Surgery Unit Università degli Studi della Campania "Luigi Vanvitelli" Naples Italy; ^2^ Otorhinolaryngology Unit Università degli Studi della Campania "Luigi Vanvitelli" Naples Italy; ^3^ Anesthesioly and Intensive Care Unit Università degli Studi della Campania "Luigi Vanvitelli" Naples Italy; ^4^ Oncology, Department of Precision Medicine Università della Campania "L. Vanvitelli" Naples Italy

**Keywords:** hypercapnia, hypoxemia, laser tube, rigid bronchoscopy, ventilation

## Abstract

**Introduction:**

Central airway tumors involving the trachea and main‐stem bronchi are a common cause of airway obstruction and a significant cause of mortality among the patients of thoracic diseases with respiratory failure. Debulking in rigid bronchoscopy is quick, safe, and effective. It can be complex and hard in patients with severe bronchial or tracheal obstruction and/or with intraluminal bleeding tumors because of inadequate distal airway control. We have used laser tube as a new technique of ventilation for severe central airway obstruction.

**Materials and Methods:**

Forty‐six patients with severe airway obstruction undergoing rigid bronchoscopy from September 2020 to June 2022 at the Thoracic Surgery Department of the University L. Vanvitelli of Naples underwent placement of laser tube.

**Results:**

In all patients who underwent rigid bronchoscopy with the use of the laser tube, a reduction of obstruction of more than 50% was obtained and in all patients no hypoxia (saturation < 88%), nor hypercapnia, nor significant bleeding were reported.

**Discussion:**

The results of this study suggest that rigid bronchoscopic debulking with the use of laser tube is a safe and effective technique in the management of central airway obstruction.

**Conclusions:**

The use of the laser tube allows the monitoring of gas exchange, which controls hypoxemia. Thanks to the double cuff put distally to the tracheal obstruction or in the contralateral bronchus to the obstructed one, the laser tube prevents the flooding of blood from debulking below the stenosis. Rigid bronchoscopy with laser tube will expand its use in the future.

## Graphical abstract

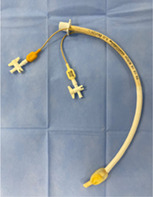

## INTRODUCTION

Central airway tumors, involving the trachea and main‐stem bronchi, are a common cause of airway obstruction and a significant cause of mortality among the patients with thoracic diseases and respiratory failure.^1^, ^2^ Surgery is the treatment of choice for patients with resectable and localized tumors, whereas radiotherapy is used for inoperable patients and postoperatively in those with incomplete resection.^3^ Rigid bronchoscopy is commonly used for patients with inoperable cancer or in those with severe airway obstruction by restoring ventilation to the collapsed lung, placing endotracheal/bronchial stents, and removing tumors. Rigid bronchoscopic debulking is quick, safe, and effective, however it can be complex and hard in patients with severe bronchial or tracheal obstruction and/or with intraluminal bleeding tumors because of inadequate distal airway control. Currently, spontaneous assisted ventilation and high‐frequency jet ventilation (HFJV) are the most usual ventilation methods. However, we have used laser tube as a new technique of ventilation for severe central airway obstruction.

## MATERIALS AND METHODS

**FIGURE 1:**
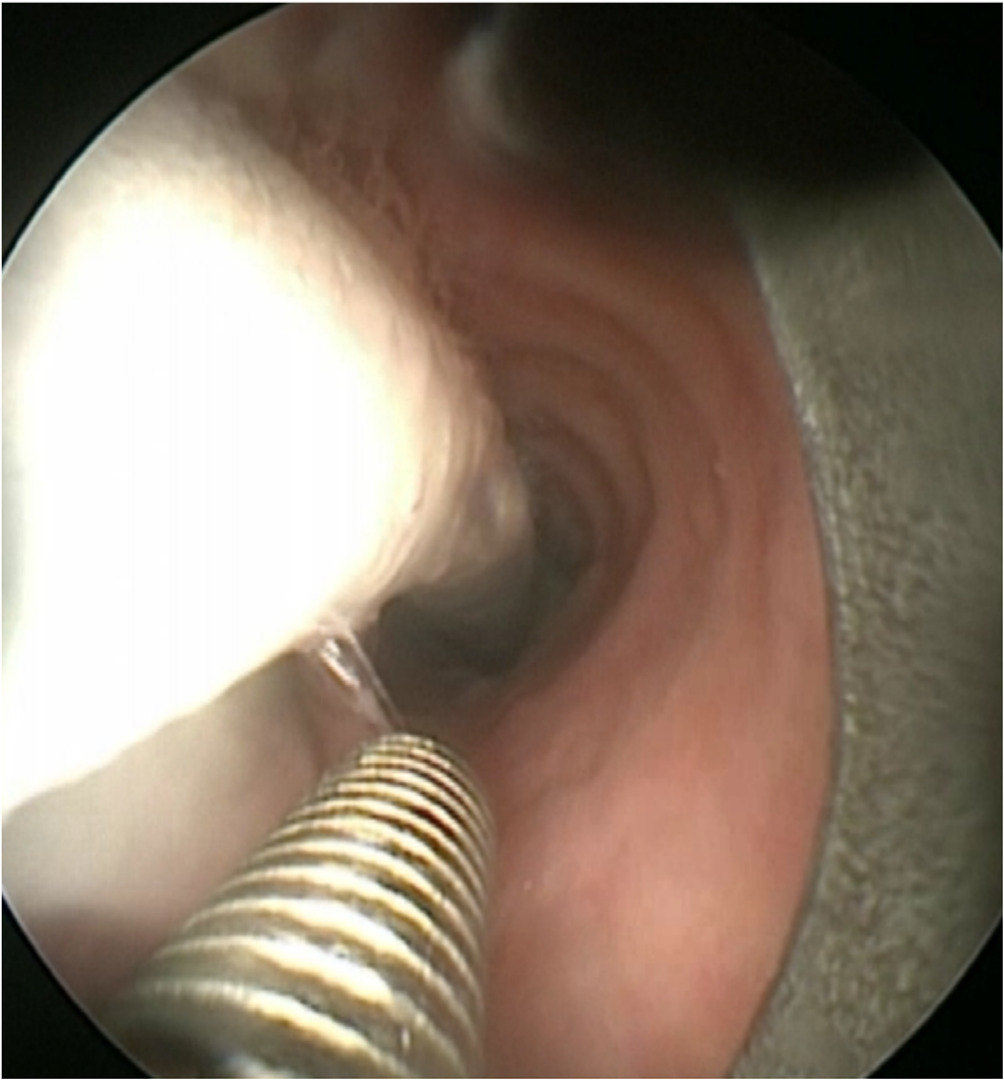
Rigid bronchoscope slides parallel along the laser tube under direct endoscopic control, guiding the positioning of the laser tube.

**FIGURE 2:**
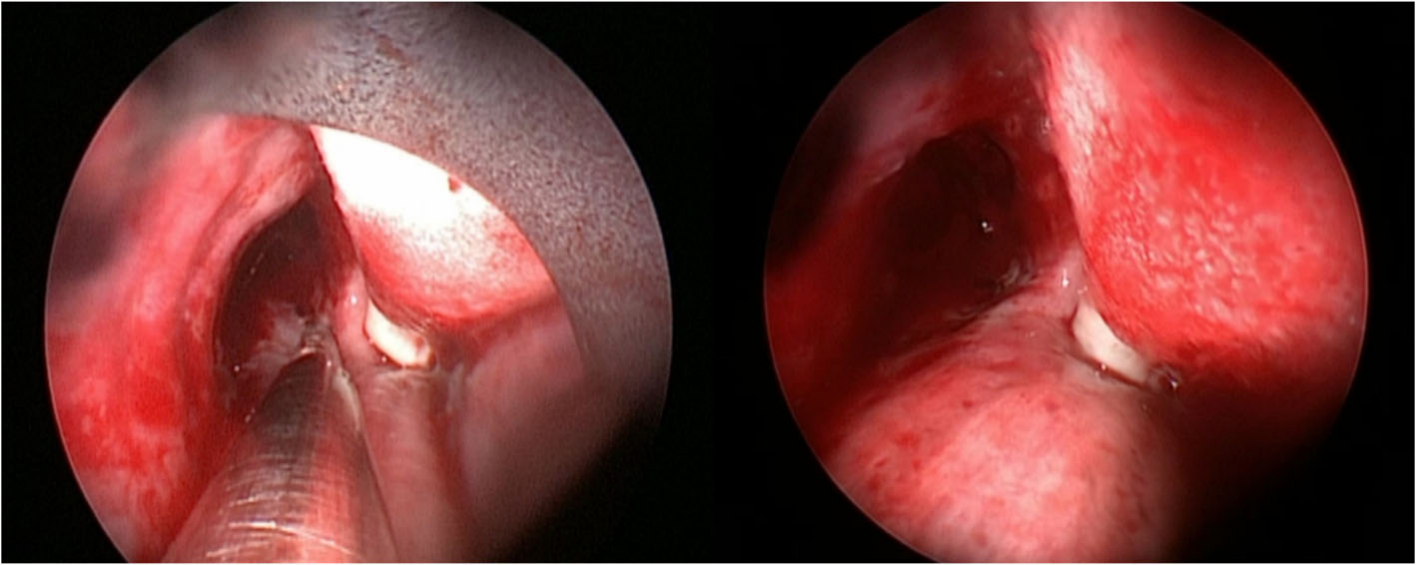
The positioning of the lase tube into the right main bronchus is easy for its anatomy

**FIGURE 3:**
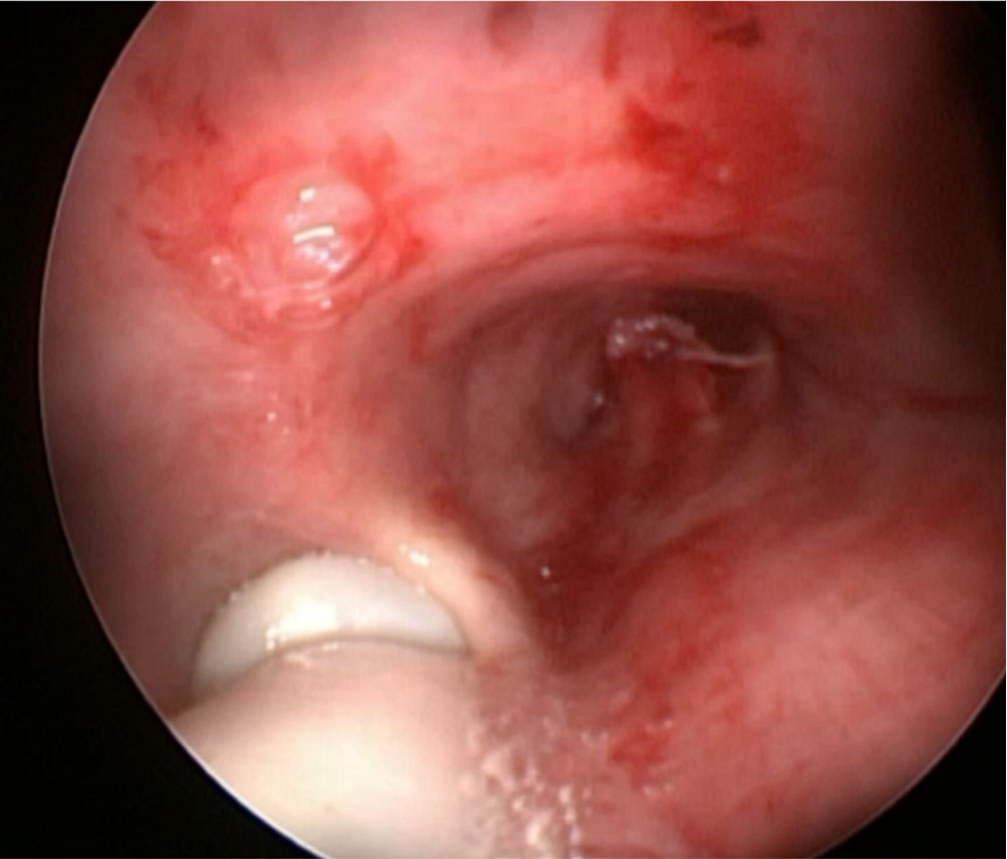
The positioning of the laser tube into the right main bronchus can be difficult. It can be guided by rigid bronchoscope, inserting it into the right bronchus and sliding the laser tube into the left bronchus; it can also be guided under fiber optic bronchoscope.

This is an observational retrospective single‐center study whose primary aim is to confirm the validity of the use of laser tube for ventilation of severe central airway obstruction in rigid bronchoscopy. From September 2020 to June 2022, 46 patients with severe airway obstruction underwent rigid bronchoscopy with the placement of laser tube at the Thoracic Surgery Department of the Vanvitelli University of Naples. Laser tube is a tracheal tube, resistant when using laser surgery. It is a sterile, for single use tube with two cuffs, one inside the other and a laser guard foil. It is 17 cm long, the outer diameter is 4 mm. It has a fixed connector. The tube is resistant to AR Laser, ND/YAG Laser, and CO_2_ Laser with a wave length ranging from 0.488 to 10.6. The tube's contraindication is a latex allergy. All patients underwent general anesthesia. Before induction, local lidocaine (2% to 4%) was nebulized through the vocal cords during laryngoscopy. The patients were oxygenated using a bag valve mask with 15 L/min with 100% oxygen so patients could keep an adequate saturation for approximately 5 minutes before intubation. All patients were anesthetized by intravenous anesthesia using: midazolam 10 mg intravenous infusion; propofol 3 mg/Kg/h intravenous infusion; Fentanest 0,2 mg/Kg/h intravenous infusion; rocuronio 0,15 mg/Kg for neuromuscular blockade. Patients with tracheal stenosis were first intubated and ventilated by endotracheal laser tube positioned under the stenosis and cuffed, whereas rigid bronchoscope 8,5 mm was located into the proximal trachea, parallel to the tube laser (Figure 1) above the obstruction, under direct visual control for tumor debulking maneuver. In patients with bronchial obstruction, the laser tube was positioned, under direct endoscopic control, into the right or left bronchus and cuffed. After intubation of the free bronchus, the rigid bronchoscope 8,5 mm can be positioned into the obstructed main bronchus, ensuring good ventilation management and avoiding blood accumulation in the contralateral bronchus. The distal positioning of the laser tube into the right main bronchus is easy for its anatomy (Figure 2). Instead the distal positioning of the laser tube into the left main bronchus can be difficult (Figure 3); it can be more easily guided with rigid bronchoscope by inserting the rigid bronchoscope into the right bronchus and sliding the laser tube into the left bronchus; or it can be guided under fiber optic bronchoscope. When bronchial or tracheal recanalization was completed, the blood and secretions accumulated between the two cuffs of the laser tube and the trachea or the contralateral bronchus to the obstructed one are then aspirated. Rigid bronchoscope is removed under direct sight. At the end of the debulking, the laser tube is positioned about 2 cm distant from the tracheal carina for adequate ventilation. Sugammadex 2 mg/kg iv is administered for awakening and then, the double cuff of the laser tube is deflated and when the patient breathes spontaneously the laser tube is removed.

### Statistical analysis

In our study, we compared the values of CO_2_ and saturation between two groups, the first group includes 46 patients undergoing rigid bronchoscopy with the use of the laser tube, and the second group of 39 patients in which the laser tube was not used. During rigid bronchoscopy, SpO_2_ in patients with tube laser was 92 ± 2.07 and CO_2_ was 42 ± 1.56, whereas in patients without laser tube, SpO_2_ was 83 ± 2.97 and CO_2_ was 83 ± 2.97. The Pearson correlation test was used to evaluate the correlation between the values of CO_2_ and saturation of the first group and of the second group. *p*‐Value <0.001 was considered statistically significant. MedCalc statistical software (version 12.3; Broekstraat 52, Mariakerke, Belgium) was used for the analysis.

## RESULTS

This is an observational retrospective single‐center study whose primary aim was to confirm the validity of the use of laser tube for ventilation of severe central airway obstruction in rigid bronchoscopy. Eighty‐five patients (51 male; 34 female; mean age, 61) with severe airway stenosis underwent rigid bronchoscopy from September 2020 to June 2022 at the Thoracic Surgery Department of the Vanvitelli University of Naples. Forty‐six patients underwent placement of laser tube. The inclusion criteria included patients with tumor in the central airways (mainstem bronchi, trachea) and presence of severe dyspnea or respiratory distress. The exclusion criteria included patients with symptoms attributable to predominant extrinsic compression. All patients underwent high‐resolution chest computed tomography (CT) to locate the tumor site and pre‐anesthetic evaluation and routine blood investigations, including coagulation profile, blood count completed, serum electrolytes, liver and renal function tests, and arterial blood gas analysis. Dyspnea was graded according Medical Research Council scale from 0 to 4. The luminal obstruction was graded as: grade 1:< 50%, grade 2: 50% to 74%, grade 3: 75% to 89% and grade 4: 90% to 100%. The histopathology findings of the tumors were: 19 adenocarcinoma (42%); 13 adenosquamous carcinoma (28%); and 14 squamous carcinoma (30%). The sides of tumors were: 13 (28%) trachea, five (11%) right main bronchus, seven (15%) left main bronchus, six (13%) upper left bronchus, nine (20%) upper right bronchus, one (2%) medium bronchus, and five (11%) right lower bronchus (table 1).

**TABLE 1:** Characteristics of patients

Patients, *n*	46
Age, years (median)	61
Sex, male (%)	31 (68)
Sex, women (%)	15 (32)
Side of tumor, *n* (%)	
Trachea	13 (28)
Right main bronchus	5 (11)
Left main bronchus	7 (11)
Left upper bronchus	6 (13)
Right upper bronchus	9 (20)
Medium bronchus	1 (2)
Right lower bronchus	5 (11)
Histology, *n* (%)	
Adenocarcinoma	19 (42)
Adenosquamous carcinoma	13 (28)
Squamous carcinoma	14 (30)

During rigid bronchoscopy, SpO_2_ in patients with laser tube was 92 ± 2.07 and CO_2_ was 42 ± 1.56, whereas in patients without tube laser, SpO_2_ was 83 ± 2.97 and CO_2_ was 83 ± 2.97 (Table 2).

**TABLE 2:** Characteristics of two study groups: pCO_2_ and SpO_2_

Variables	All patients (*n* = 85)	With tube group (*n* = 46)	Without tube group (*n* = 39)	*p‐*value
FEV1 (%)	68	68	69	0.45
SpO_2_ (median)	89	92	83	<0.001
pCO_2_ (median)	45	42	51	<0.001

The Pearson correlation test was used to evaluate the correlation between the values of CO2 and saturation of the first group and of the second group and *p*‐value <0.001 was considered statistically significant. Therefore, the use of the laser tube reduces the risk of admission to postoperative intensive care. No complications (laryngeal, oropharyngeal, tracheal‐bronchi perforation, and vocal cord injury,) were reported. The results of this study suggest that rigid bronchoscopic debulking with the use of laser tube is a safe and effective technique in management of central airway obstruction.

## DISCUSSION

**FIGURE 4:**
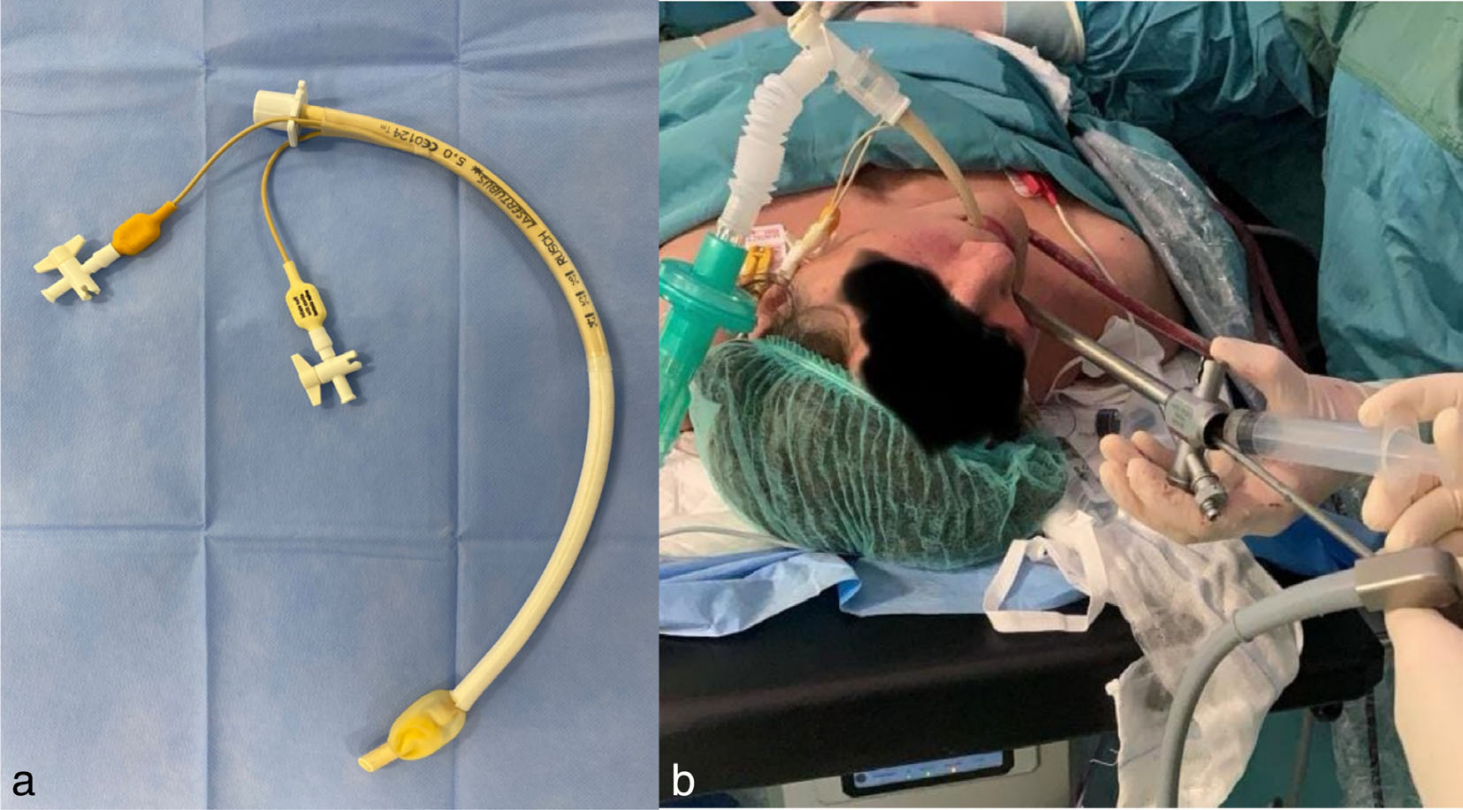
(a) Laser tube is a tracheal tube, laser resistant: two cuffs, one inside the other; laser guard foil; 17 cm long., 4 mm outer diameter, fixed connector, sterile, for single use. (b) Rigid bronchoscopy remains a “ventilation challenge” because of the intrinsic nature of the surgical procedure, which occupies the same channel through which the anesthetist ventilates the patient.

Rigid bronchoscopy is used as a palliative treatment for patients with severe obstructions in the trachea and main bronchi. It can alleviate symptoms with stent placement, laser ablation, and other techniques in many patients with a life‐threatening airway obstruction.^4^, ^5^, ^6^, ^7^ Rigid bronchoscopy is routinely performed during general anesthesia, because the insertion of an airway device needs muscle relaxation and ventilation control is necessary.^8^, ^9^, ^10^ In some patients, ventilation and oxygenation is challenging because the airway caliber is compromised by airway narrowing, obstruction, and by the endoscopic instrumentation. Accurate respiratory monitoring is vital to good outcomes. Laser tube in rigid bronchoscopy is mainly used in patients with severe bronchial and tracheal obstruction when the respiratory space is severely reduced by the tumor, making ventilation with rigid bronchoscopy distal to the stenosis challenging.^11^, ^12^, ^13^ In rigid bronchoscopy, spontaneous assisted ventilation and HFJV are two most frequently methods of ventilation.^14^ Spontaneous assisted ventilation is not always possible in patients with severe airway obstruction, whereas HFJV is the chosen ventilation method in such patients.  HFJV can determine barotrauma and hypercapnia because the gas egress is not ensured.^15^, ^16^ Puma et al.^17^ have used tracheal tube supplied in the Fantoni translaryngeal tracheotomy kit for better control of the airway below the stenosis and safer anesthesia management. We used laser tube that, thanks to laser guard foil, is resistant to the AR Laser, ND/YAG Laser, and CO2 Laser. Although the diameter of the laser tube is small, it guarantees adequate ventilation thanks to its double cuff, which can be positioned both at the tracheal and bronchial level. The use of the laser tube allows to monitor gas exchange, controlling hypoxemia (arterial saturation <90%) and hypercapnia, both signs of ventilatory dysfunction.^18^ The insertion of the laser tube, thanks to the double cuff distally to the tracheal obstruction and in the contralateral bronchus to the obstructed bronchus, prevents the flooding of blood from debulking below the obstruction. The obstruction can be a life‐threatening emergency requiring prompt treatment to avoid significant hypoxia or death.^19^ Therefore, the rigid bronchoscope is used exclusively as a working channel. In all patients that underwent rigid bronchoscopy with the use of the laser tube, a reduction of obstruction of more than 50% was obtained, and in all patients no hypoxia (saturation <88%), nor hypercapnia, nor significant bleeding, nor admission in intensive care were reported. Therefore, the use of the laser tube reduces the risk of admission to postoperative intensive care.^20^ For specialists in anesthesiology and thoracic surgeons, rigid bronchoscopy remains a “ventilation challenge” owing to the intrinsic nature of the surgical procedure, which occupies the same channel through which the anesthetist ventilates the patient (Figure 4(a),(b)). Good management of patients with tracheal or bronchial obstruction involves knowledge of anatomy and techniques, a quick decision‐making process, and an understanding between the thoracic surgeon and an experienced anesthetist.

## CONCLUSION

Rigid bronchoscopy with mechanical debulking is an effective and safe modality for the palliative treatment of neoplastic airway obstruction. Anesthesia for rigid bronchoscopy is challenging, because patients undergoing this procedure have critical respiratory illnesses and have varying degrees of dyspnea or respiratory distress. In rigid bronchoscopy, anesthetists and thoracic surgeons must deal with airway management and maintenance of patients' oxygen levels. Interventional bronchoscopy is rapidly expanding, and anesthetists need to be updated on related techniques. The thoracic surgeon is generally not free from these complications.^21^ The thoracic surgeon must be continually updated with airway control and respiratory mechanics through the endotracheal tube. Anesthesia is a point of particular emphasis in the treatment of central airway obstruction.^22^, ^23^ Therefore, empathy and communication between anesthetist and surgeon is a key principle. During the induction and positioning of the laser tube, the surgeon must be present with all the necessary operating equipment. The use of the laser tube allows monitoring of gas exchange, which controls hypoxemia (arterial saturation <90%) and hypercapnia, both signs of ventilatory dysfunction. The insertion of the laser tube, thanks to the double cuff distally to the tracheal stenosis and in the contralateral bronchus to the obstructed bronchus, prevents the flooding of blood from debulking below the stenosis. Rigid bronchoscopy with laser tube will expand its use in the future, having greater importance with the improvement in additional procedures, which will be more and more effective.

## LIMITATIONS

The study is a preliminary study that needs further cases to corroborate our data.

## AUTHOR CONTRIBUTIONS

Concept and design, Gaetana Messina, Mary Bove, Anne Rainone, and Giuseppe Vicario. Administrative support, Fortunato Ciardiello, and Morena Fasano. Provision of study materials or patients, Alfonso Fiorelli, Giuseppe Vicario, and Mario Santini. Collection and assembly of data, Mario Pirozzi, Carminia Maria Della Corte, Marianna Caterino, and Beatrice Leonardi. Data analysis and interpretation, Giovanni Natale, Giorgia Opromolla, Vincenzo Di Filippo, and Eva Massimilla. Manuscript writing: all authors. Final approval of manuscript: all authors.

## CONFLICT OF INTEREST

All authors have completed the ICMJE uniform disclosure form. The authors have no conflicts of interest to declare.

## DISCLOSURE STATEMENT

The authors declare nothing to disclose.

## ETHICAL STATEMENT

The authors are accountable for all aspects of the work in ensuring that questions related to the accuracy or integrity of any part of the work are appropriately investigated and resolved. The study was led in compliance with the principles of the Declaration of Helsinki; written informed consent was obtained from all participants during preoperative communication and the protocol was approved by the Ethics Committee of the University of “Luigi Vanvitelli” of Naples n 280 on May 16, 2020.
